# Attitudes of Chinese maternal and child health professionals toward termination of pregnancy for fetal anomaly: a cross-sectional survey

**DOI:** 10.3389/fpubh.2023.1189266

**Published:** 2023-09-07

**Authors:** Ying Wu, Yanlin Liu, Xiaomin Wang, Yuqiong Zhong, Xin Zhang, Dan Luo, Xing Liu

**Affiliations:** ^1^School of Humanities, Central South University, Changsha, Hunan, China; ^2^School of Health Sciences, College of Medicine, Nursing and Health Sciences, University of Galway, Galway, Ireland; ^3^Department of Social Medicine and Health Management, Xiangya School of Public Health, Central South University, Changsha, Hunan, China; ^4^Center for Clinical Pharmacology, The Third Xiangya Hospital of Central South University, Changsha, Hunan, China; ^5^Medical Humanities Research Center, Central South University, Changsha, Hunan, China; ^6^Xiangya Hospital, Central South University, Changsha, Hunan, China; ^7^Medical Ethics Committee, Xiangya Hospital of Central South University, Changsha, Hunan, China

**Keywords:** attitudes, birth defect, termination of pregnancy for fetal anomaly, maternal and child health professional, China

## Abstract

**Objectives:**

This study explores the attitudes of Chinese maternal and child health professionals toward the termination of pregnancy for fetal anomaly (TOPFA) based on four case scenarios and further identifies the factors that influence their attitudes.

**Methods:**

This cross-sectional study, conducted from February 14–21, 2022, aimed to explore the attitudes of maternal and child health professionals toward TOPFA in Hunan Province. We targeted health service institutions across 14 prefecture-level cities and the autonomous prefecture. A questionnaire was made available online and shared via the instant communication platform, WeChat. Participants were recruited through the same platform and completed the survey online. Descriptive statistics were used to analyze the data, and binary logistic regression was performed to determine factors affecting the health professionals’ attitudes toward TOPFA, expressed as the odds ratio (OR) and 95% confidence intervals (CI).

**Results:**

The study found that 63.5% of health professionals approved of the birth of a fetus with cleft lip and palate, while 36.5% opposed it. Similarly, 39.7% approved of the birth of a fetus with phenylketonuria, while 60.3% opposed it. The percentages of those in favor of and against the birth of a fetus with precocious heart disease were 45.5 and 54.5%, respectively, and those for and against the birth of a fetus with missing fingers were 50.8 and 49.2%, respectively. The top three factors considered by health professionals when agreeing on TOPFA were “the impact of fetal disease on fetal function and growth,” “the severity of fetal disease,” and “the assessment of indications for fetal disease by professionals and related professional advice.” The majority of health professionals (75–78%) preferred joint decision-making by parents regarding the right to decide TOPFA.

**Conclusion:**

Our study indicates that the attitudes of health professionals toward TOPFA can differ significantly depending on the specific birth defect under consideration. Notably, the majority of health professionals prioritized “the impact of fetal abnormalities on fetal function and development” when deciding their support for TOPFA, advocating for the decision to be a joint one between the parents. Additionally, factors such as religious beliefs, professional training, age, and job title appeared to influence these attitudes toward TOPFA. Our findings could serve as a reference point in the development of guidelines for the prevention and management of birth defects.

## 1. Introduction

Major birth defects represent a significant public health concern that is receiving growing global attention (1). According to the World Health Organization (WHO), the global incidence of birth defects is 6.42% in low-income countries, 5.57% in middle-income countries, and 4.72% in high-income countries (2). In 2012, the Ministry of Health of the People’s Republic of China released a national report on birth defect prevention, which estimated that the incidence of birth defects in China was approximately 5.6% (2). The incidence of birth defects in China is comparable to the world average for middle-income countries (2). Addressing the global challenge of birth defects, the WHO has proposed a comprehensive strategy, inclusive of intervention strategies related to pregnancy care (3). Aligning with the WHO’s strategy, the Chinese government has implemented a systematic policy and program to counter birth defects (4).

Pregnancy presents numerous physiological challenges (5). Consequently, prenatal care, defined as the healthcare a woman receives during pregnancy, is crucial. Early and regular prenatal visits with a healthcare provider significantly contribute to the health of both the mother and the fetus (6). Prenatal diagnosis is a process used to evaluate the presence of disease or potential disease in the fetus (7). In instances where birth defects are associated with a poor prognosis, parents may opt for the termination of the pregnancy (8). One study showed that the incidence of pregnancy termination following prenatal diagnosis was high in Hunan Province, China (9). Concurrently, physicians with critical dilemmas regarding pregnancy termination in cases of mild congenital abnormalities (10).

Termination of pregnancy is a commonly used health intervention (11), and it is widely accepted that the procedure requires careful consideration (12, 13). Over the past 30 years, more than 60 countries have liberalized their laws on termination of pregnancy (14–17). In China, termination of pregnancy for fetal abnormalities (TOPFA) is permitted in maternal and child care settings, physicians are required to explain the situation to both partners and provide medical advice on whether to terminate the pregnancy. TOPFA has become more complex and pervasive for maternal and child health professionals (18). Several studies have examined the attitudes of health professionals toward the TOPFA in different countries, including France (19), England (20), Belgium (21), Israel (18), and Turkey (22). However, there are notable cross-cultural variations in the practice of prenatal diagnosis and TOPFA (19). Sociodemographic characteristics of different populations can significantly influence their attitudes toward the termination of pregnancy following the detection of fetal abnormalities (23, 24). Health professionals’ opinions and attitudes are shaped by diverse cultural backgrounds (22).

Understanding the health professionals’ attitude toward TOPFA is crucial to providing high-quality care and support to individuals making the most appropriate decision on the baby’s birth defect, and it can provide insights into the concept of living with a disability from a different perspective (25). However, there remains a lack of empirical research on Chinese maternal and child health professionals’ attitudes toward TOPFA. Our study aimed to investigate the attitudes of health professionals toward TOPFA and to further analyze the relevant factors that influence these attitudes.

## 2. Methods

### 2.1. Study design

The study employed a cross-sectional design to explore the attitudes of maternal and child health professionals toward TOPFA. The sample consisted of professionals actively engaged in the prevention and treatment of birth defects across 14 prefecture-level cities and the autonomous prefecture in Hunan Province.

### 2.2. Participants

Maternal and child health professionals across 14 prefecture-level cities and the autonomous prefecture in Hunan Province, who met the study’s inclusion criteria, were recruited for participation in the survey. The participants from clinical services (gynecology, obstetrics, pediatrics, genetic counseling, radiology, assisted reproduction), maternal and childcare services (health department, women’s health, child health, premarital/pre-pregnancy care, health education and information), and other specialties related to maternal and child health services. The inclusion criteria required that participants were (1) at least 18 years of age, (2) qualified professionals employed in medical institutions and maternal and child health institutions, and (3) willing to participate in this study after giving informed consent. Informed consent was obtained from all survey participants.

### 2.3. Questionnaire

We utilized an online, self-designed, and case-based questionnaire, consisting of two parts: sociodemographic information, and a case-based attitude questionnaire on TOPFA (see the Supplementary Appendix for details of the questionnaire). The initial section covered sociodemographic data such as participant age, gender, ethnicity, residence, educational background, marital status, religious affiliation, professional category, and working unit type.

The second section aimed to gauge participant attitudes toward TOPFA, developed from insights garnered from a preceding qualitative study. This study incorporated the participation of 30 birth defects prevention professionals and yielded 11 TOPFA cases. These cases were subsequently revised and incorporated into the attitude-oriented questionnaire. Two rounds of expert consultation with eight experts were undertaken to refine and augment the questionnaire based on their feedback. Four distinct fetal anomalies were eventually highlighted in the case-based examples: congenital heart disease, cleft lip and palate, digit amputation, and phenylketonuria. Selection was predicated on the anomaly type (visible versus non-visible defects) and the complexity in accurately determining the severity given present diagnostic capabilities, which resulted in uncertain prognoses for post-birth outcomes.

Based on the four cases, the questionnaire addressed three main questions. First, participants were asked whether they agreed with TOPFA. Four case scenarios involving fetuses with varying degrees of congenital defects were presented, and participants were asked to decide on TOPFA. Response options were set as “agree” and “disagree.”

Second, we sought to understand the factors that health professionals considered when deciding whether agreed with TOPFA. Multiple choice was used to elicit participants’ views on the consideration factors in the four cases.

Third, we investigated who in the family health professionals believed had the final decision-making authority in TOPFA. The questionnaire presented family roles in the four cases and asked participants about their attitudes toward the viewpoints of each given role and who they believed had the right to make the final decision. Attitudinal questions were assessed on a 5-point Likert scale (“strongly agree,” “agree,” “uncertain,” “disagree,” and “strongly disagree”). Table 1 provides an overview of the key characteristics of the four case scenarios.

**Table 1:** Characteristics of the four cases.

	Case 1 (congenital heart disease)	Case 2 (cleft lip and palate)	Case 3 (finger loss)	Case 4 (phenylketonuria)
Characteristics of disease	Multiple surgeries required to repair Prospect of cure High cost of surgery	Can be surgically repaired Difficult to judge the recovery effect	Absence of three fingers Inability to assess prognostic outcome	May lead to growth retardation and neuropsychiatric problems in children Can grow and develop normally and maintain a normal life if diagnosed and treated early Requires lifelong treatment through dietary control and medication High economic pressure
Gestational age	23 weeks of gestation (6 months)	22–24 weeks of gestation (about 6 months)	18 weeks of gestation (more than 4 months)	16 weeks of gestation (4 months)
Family situation	Had terminated a previous pregnancy for fetal anomaly and therefore the family cherishes this fetus	Parents and grandparents of the fetus have different views on termination of pregnancy for fetal anomaly Family members fear discrimination after the birth of the fetus	Parents and grandparents of the fetus have different views on termination of pregnancy for fetal anomaly Fear that the child will be permanently disabled and discriminated against	Fetal parents have different views on the termination of pregnancy for fetal anomaly
Views of different roles	The parents intend to keep the fetus	The parents were willing to continue the pregnancy, but the grandparents were not	The parents wanted to terminate the pregnancy, but the grandparents thought the pregnancy could continue	The mother wanted to continue the pregnancy, but the father wanted to terminate it

Regarding the retest reliability, Cronbach’s alpha yielded a score of 0.758, indicating a satisfactory level of internal consistency. Content validity was evaluated by eight independent experts in the fields of birth defect, medical ethics and public health. After the questionnaire’s initial draft was composed, a preliminary survey was carried out with 12 health professionals to obtain their feedback on the content. In response to their recommendations, revisions were made in the phrasing of the questionnaire, culminating in the final version.

### 2.4. Data collection

The Hunan Province regularly conducts provincial training activities concerning birth defect prevention and control. As a part of these activities, specialized WeChat groups have been established. WeChat is a widely used instant communication application in China, and members of this group include professionals from 14 distinct prefecture-level cities and the autonomous prefecture in Hunan Province, all operating in birth defect prevention-related fields. Each member of the group was invited to participate in the study. The data was gathered using an online questionnaire, distributed via a link to the WeChat group.

The enrollment period spanned from February 14th to 21st, 2022. A total of 785 responses were collected, out of which 750 were deemed valid after the exclusion of incomplete or missing submissions. Following the signing of consent forms, the survey continued for those participants who wished to further participate.

### 2.5. Data analysis

The data was organized using Microsoft Excel and analyzed using SPSS 26.0. Descriptive statistics, including frequency and composition ratios, were used to summarize the characteristics of health professionals and their attitudes toward TOPFA. For simplified descriptive statistics the categories “strongly disagree” and “disagree” were summarized as disagreement while “agree” and “strongly agree” were summarized as agreement. Binary logistic regression analysis was employed to determine factors affecting health professionals’ attitudes toward TOPFA. The dependent variable was defined as health professionals’ attitudes toward TOPFA, with a value of 0 indicating “agree” and a value of 1 indicating “disagree.” The results of the binary logistic regression analysis were expressed in odds ratios with a 95% confidence interval, and the significance level was set at 0.05 on both sides.

### 2.6. Ethics statement

The study involved human participants and was reviewed and approved by the Institutional Review Board (IRB) of Xiangya School of Public Health at Central South University (registration number XYGW-2022-97). Informed consent for participation was provided through the completion of the survey. The study was conducted in compliance with the principles outlined in the Declaration of Helsinki.

## 3. Results

### 3.1. Sociodemographic characteristics

The study included a predominantly female population, with 79.9% being women. The majority of participants were between the ages of 31–50 years (60.5%), and the ethnic group with the highest representation was Han (69.2%). The majority of participants resided in urban areas (88.3%) and were highly educated, with 92.3% having attained a college degree or higher. Most participants were married (72.1%), while only a small proportion reported having a religious affiliation (1.3%). Among the study sample, 69.9% reported having children, while 26.8% reported having close relatives or friends who had children with birth defects. The majority of participants worked in maternal and child-related medical institutions (68.6%), with over half working in clinical service departments (55.2%). A minority of participants reported receiving training related to birth defects prevention and control (35.1%), and even fewer had received ethics training related to birth defects (25.3%) (see Table 2).

**Table 2:** Characteristics of participants (*N* = 750).

Variables		Number (N)	Percentage (%)
Gender			
	Male	151	20.1
	Female	599	79.9
Age (years)			
	≤30	258	34.4
	31–50	454	60.5
	≥51	38	5.1
Ethnicity			
	Han	519	69.2
	Minority	231	30.8
Current residence			
	Urban (including counties and towns)	662	88.3
	Rural	88	11.7
Educational background			
	High school and below (including secondary school)	59	7.8
	Junior college and undergraduate college	620	82.7
	Master’s and above	71	9.5
Marital status			
	Unmarried	188	25.1
	Married	541	72.1
	Other (divorced, widowed)	21	2.8
Religious affiliation			
	Yes	10	1.3
	No	740	98.7
Children			
	Yes	524	69.9
	No	226	30.1
Relatives/friends with children with birth defects			
	Yes	201	26.8
	No	549	73.2
Unit type			
	Maternal and child-related medical and health institution ^a^	514	68.6
	General hospital	154	20.5
	Other (community health service centers, township hospital)	82	10.9
Professional title			
	Primary-level professional title	228	30.4
	Intermediate-level professional title	276	36.8
	High-level professional title	140	18.7
	None	106	14.1
Type of work			
	Full-time	653	87.1
	Part-time	97	12.9
Participants (multiple choice)			
	Clinical service departments ^b^	475	55.2
	Maternal and child health sector ^c^	279	32.4
	Other ^d^	107	12.4
Received training related to birth defect prevention and control in the last 3 years			
	Yes	263	35.1
	No	487	64.9
Received training on ethics related to birth defects in the last 3 years			
	Yes	190	25.3
	No	560	74.7

a Maternal and child-related medical institutions refer to institutions that specialize in maternal and child health care institutions, including maternal and child health stations and hospitals.

b Clinical service departments include gynecology, obstetrics, pediatrics, genetic counseling, radiology, and assisted reproduction.

c Maternal and child health departments encompass the health department, women’s health, child health, premarital/preconception health, and health education and information sections.

d Other refers to participants in professions related to maternal and child health services not included in the above categories.

### 3.2. Attitudes of health professionals toward TOPFA

**Figure 1:**
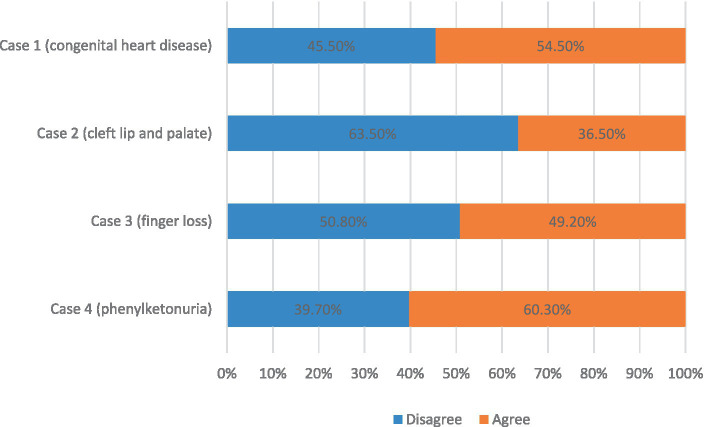
Attitudes of health professionals toward termination of pregnancy for fetal abnormalities (TOPFA).

Survey data revealed minor disparities in health professionals’ attitudes toward termination of pregnancy in cases of fetal congenital heart disease or finger loss. Notably, 45.5% of the surveyed professionals objected to pregnancy termination for a fetus with congenital heart disease, in contrast to the 54.5% who were in agreement. A comparable dichotomy was observed for cases of fetal finger loss, where 50.8% of professionals opposed termination, while 49.2% supported it. However, attitudes markedly varied concerning fetuses with cleft lip and palate or phenylketonuria. In cases of cleft lip and palate, 63.5% of health professionals opposed termination, with only 36.5% in agreement. Conversely, for phenylketonuria, 39.7% of professionals opposed termination, while a majority of 60.3% supported it. These results are presented in Figure 1.

### 3.3. Consideration of health professionals on TOPFA

A comprehensive analysis of the four cases of birth defects showed a convergence of factors considered by maternal and child health professionals. For these four cases, the three most frequently selected factors by professionals out of 11 were “the impact of fetal disease on fetal function and growth,” “the severity of fetal disease,” and “the assessment of indications for fetal disease by professionals and related professional advice.” As shown in Table 3.

**Table 3:** Health professionals’ considerations on TOPFA.

Factors considered by health professionals (Multiple choice)	Case 1 Congenital heart disease	Case 2 Cleft lip and palate	Case 3 Finger loss	Case 4 Phenylketonuria
	Number (N)	Number (N)	Number (N)	Number (N)
All health professionals	*N* = 750	*N* = 750	*N* = 750	*N* = 750
The effect of fetal autoimmune diseases on fetal body function and growth and development	496	473	497	528
The severity of fetal diseases	462	460	477	499
The independent choice of the pregnant woman and her family	359	369	358	350
Evaluation of indications for treatment of fetal diseases by clinical professionals and related professional advice	403	386	374	378
Health professionals who disagree with TOPFA	*n* = 341	*n* = 476	*n* = 381	*n* = 298
The effect of fetal autoimmune diseases on fetal body function and growth and development	207	281	224	179
The severity of fetal diseases	204	291	235	184
The independent choice of the pregnant woman and her family	198	263	196	140
Evaluation of indications for treatment of fetal diseases by clinical professionals and related professional advice	209	263	195	159
Health professionals who agree with TOPFA	*n* = 409	*n* = 247	*n* = 369	*n* = 452
The effect of fetal autoimmune diseases on fetal body function and growth and development	289	192	273	349
The severity of the fetal disease	258	169	242	315
The possible negative impact of the fetal disease on the family or a family member	227	133	193	249
Assessment of the indications for treatment of fetal diseases by clinical professionals and related professional advice	194	123	179	219

SD, Standard deviation.

In cases where health professionals disagreed with TOPFA, the top three most frequently selected factors were similar to those of all professionals, with only a slight variation in ranking. Conversely, for those who agreed with TOPFA, the top two most frequently selected factors in each case were consistent with those chosen by all professionals. However, the third most important and frequently considered factor differed, namely, “the possible negative impact of the fetus’ illness on the family or a family member.”

### 3.4. Attitudes of health professionals toward family member’s decision-making rights on TOPFA

#### 3.4.1. Attitudes of health professionals toward the different roles in each scenario

The attitudes of professionals toward who should make the final decision regarding the birth of a fetus with a birth defect varied across the four scenarios, as shown in Table 4. In the case of congenital heart disease, 321 (42.8%) professionals agreed with the idea of parents’ decision to keep the child, while 291 (38.8%) disagreed. Notably, in the case of cleft lip and palate, 401 (53.5%) professionals agreed with the idea of parents’ decision to keep the child, while 206 (27.5%) disagreed. Regarding grandparents having the right to decide whether the child should be born: 193 (25.7%) agree, while 395 (52.7%) disagree. In the case of finger loss, 331 (44.1%) professionals agreed with the idea of parents being able to decide whether to terminate the pregnancy, while 249 (33.2%) disagreed. But there was an equal number of professionals who agreed and disagreed with the idea of grandparents having the right to decide whether the child should be born: 272 (36.3%) agreed, while 283 (37.7%) disagreed. In the case of phenylketonuria, 352 (46.9%) professionals agreed with the father’s decision not to want the child, while 213 (28.4%) disagreed. Regarding the mother’s decision to keep the child, 237 (31.6%) professionals agreed and 359 (47.9%) disagreed.

**Table 4:** Attitudes of health professionals regarding the different role in each scenario.

	Ideas of different roles in each case	Agree	Uncertain	Disagree
Case 1 (Congenital heart disease)	The parents want to keep the baby	321 (42.8%)	138 (18.4%)	291 (38.8%)
Case 2 (Cleft lip and palate)	The parents want to keep the baby	401 (53.5%)	143 (19%)	206 (27.5%)
	The grandparents wanted the mother to have an abortion	193 (25.7%)	162 (21.6%)	395 (52.7%)
Case 3 (Finger loss)	The parents want to give up the baby	331 (44.1%)	170 (22.7%)	249 (33.2%)
	The grandparents thought the baby could be born	272 (36.3%)	195 (26%)	283 (37.7%)
Case 4 (Phenylketonuria)	The mother decided to keep the baby	237 (31.6%)	154 (20.5%)	359 (47.9%)
	The father does not want the baby	352 (46.9%)	185 (24.7%)	213 (28.4%)

#### 3.4.2. Health professionals’ attitudes toward final decision-making authority

**Figure 2:**
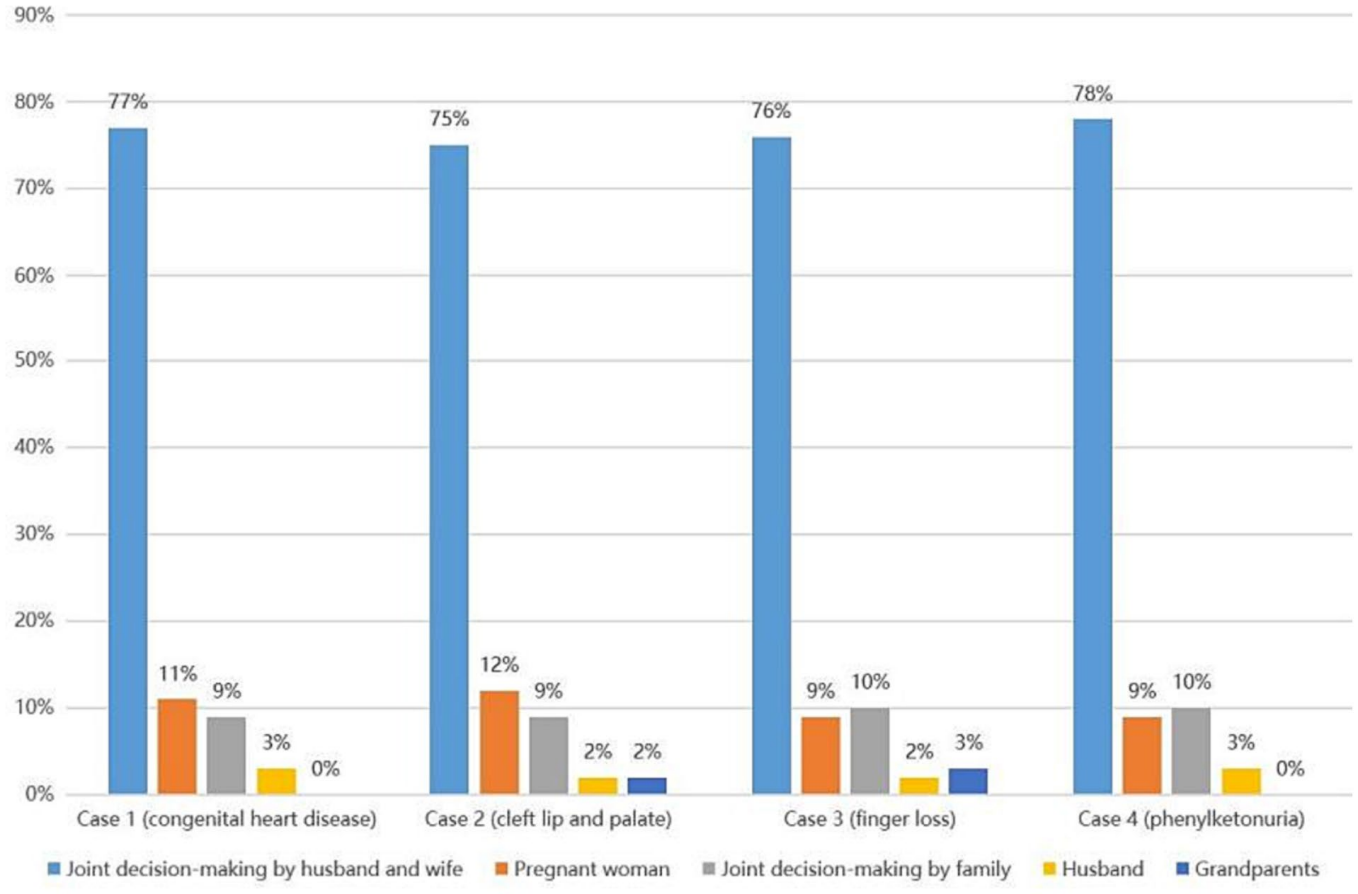
Health professionals’ attitudes toward final decision-making authority on TOPFA.

Health professionals were more consistent in their selection of final decision-making authority across the four types of birth defects. The majority of professionals (75–78%) preferred the option of “joint decision-making by husband and wife,” followed by the options of “pregnant woman” (9–12%) and “joint decision-making by family (all family members)” (9–10%). A minority chose “husband” (2–3%) and “grandparents” (0–3%), as shown in Figure 2.

### 3.5. Factors influencing the health professionals’ attitudes toward TOPFA

In order to determine factors influencing health professionals’ attitudes toward TOPFA, binary logistic regression analysis was employed. The results of this analysis are presented in Table 5, which outlines the factors influencing the health professionals’ attitudes.

**Table 5:** Factors affecting the health professionals’ attitudes toward TOPFA.

Covariates	Categories	Odds ratio	B	SE	Waid *X*^2^	*P*-values	95% CI
Case 1 (Congenital heart disease)							
Religious or not							
	Yes	5.52	1.71	0.81	4.49	0.034*	1.14–26.85
	No (ref.)						
Received training related to birth defect prevention and control in the last 3 years							
	Yes	1.79	0.58	0.24	6.01	0.014*	1.12–2.85
	No (ref.)						
Case 2 (Cleft lip and palate)							
Age (years)							
	≤30 (ref.)						
	31–50	1.24	0.21	0.23	0.83	0.362	0.78–1.95
	≥51	2.40	0.87	0.44	3.88	0.049*	1.00–5.72
Type of work							
	Full-time (ref.)						
	Part-time	0.39	−0.95	0.28	11.93	0.001*	0.22–0.66
Professional title							
	None (ref.)						
	Primary-level professional title	0.63	−0.46	0.29	2.49	0.114	0.35–1.12
	Intermediate-level professional title	0.50	−0.69	0.31	4.97	0.026*	0.27–0.92
	High-level professional title	0.72	−0.32	0.34	0.91	0.341	0.37–1.41
Case 4 (Phenylketonuria)							
Ethnicity							
	Han Chinese (ref.)						
	Ethnic minority	0.68	−0.39	0.19	4.41	0.036*	0.47–0.98
Marital status							
	Unmarried (ref.)						
	Married	0.54	−0.62	0.26	5.65	0.017*	0.32–0.90
	Others (divorced, widowed)	0.60	−0.51	0.52	0.94	0.333	0.22–1.68
Clinical service departments							
	Yes	1.42	0.35	0.18	3.92	0.048*	1.00–2.01
	No (ref.)						
Maternal and child health sector							
	Yes	1.40	0.33	0.16	4.35	0.037*	1.02–1.91
	No (ref.)						

*Statistical significance at 5% level.

In terms of sociodemographic characteristics, our study found that religious health professionals (OR = 5.52; 95% CI = 1.14–26.85) and those who had received birth defect prevention and control-related training (OR = 1.79; 95% CI = 1.12–2.85) were more likely to oppose the termination of pregnancy of the fetus with congenital heart disease (Case 1). For cleft lip and palate (Case 2), health professionals aged 51 years and older (OR = 2.40; 95% CI = 1.00–5.72) were more likely to oppose the termination of pregnancy of the fetus. Part-time professionals (OR = 0.39; 95% CI = 0.22–0.66) and health professionals with intermediate-level professional titles (OR = 0.50; 95% CI = 0.27–0.92) were more likely to support the termination of pregnancy of the fetus. For phenylketonuria (Case 4), health professionals in clinical services (OR = 1.42; 95% CI = 1.00–2.01) and maternal and child health departments (OR = 1.40; 95% CI = 1.02–1.91) preferred to oppose the termination of pregnancy of the fetus, while professionals who are minorities (OR = 0.68; 95% CI = 0.47–0.98) and married (OR = 0.54; 95% CI = 0.32–0.90) were more likely to support the termination of pregnancy of the fetus.

## 4. Discussion

### 4.1. Attitudes of health professionals toward TOPFA

The study revealed diverse attitudes among health professionals toward TOPFA, contingent on the nature of the birth defect. Over 50% of maternal and child health practitioners endorsed termination in instances of congenital heart disease and phenylketonuria. The diagnostic journey, decision-making process, and subsequent management of critical congenital heart disease introduce a multifaceted challenge for clinicians and families (26). Particularly, the diagnosis and treatment of a critically ill congenital heart disease child significantly impact parents’ health and wellbeing, and the experience is profoundly stressful and transformative for families (26). Regarding phenylketonuria, despite the availability of clear treatment options, the prognosis varies, and continuous treatment from the neonatal period, with lifelong treatment recommended (27). Children over the age of five score poorly in all quality of life domains, and dietary management of PKU places a financial burden on nearly two-thirds of families with affected children (28). Parents of children with PKU also experience significant stress raising their children (29). These factors may contribute to health professionals being concerned about the prognosis and quality of life of fetuses with incurable, lifelong treatment for this inherited metabolic disease.

Over half of the surveyed maternal and child health professionals did not support the termination of pregnancy in instances of Case 2 (cleft lip and palate). Cleft lip with or without cleft palate is one of the most common birth defects (30), and with the advancement of medical technology, most children who receive treatment for these conditions recover well and lead healthy lives (30). Health professionals are more optimistic about the prognosis and future growth and development of fetuses with postnatal repairable structural malformations than in fetuses with postnatal irreparable or difficult-to-repair structural malformations.

The sentiments of maternal and child health professionals exhibited a degree of uncertainty in the case of finger loss, with those opposing the termination of pregnancy exceeding those in favor by a mere 1.6%. This contradicts a Turkish study that found a higher incidence of health professionals rejecting termination requests for pregnancies with limb abnormalities. The prevailing perception among Turkish physicians is that termination is morally indefensible as they believe it only slightly impairs the child’s autonomy (22). Our findings correspond closely with a French study, in which abnormalities such as missing limbs are not considered severe fetal abnormalities, thus complicating the decision-making process surrounding pregnancy termination (10).

### 4.2. Consideration of health professionals on TOPFA

Our findings show that health professionals are very consistent in the factors they consider when deciding whether to support TOPFA. These main consideration factors included “the impact of fetal anomaly on fetal function and development,” “the severity of fetal anomaly,” “professional assessment of the indication for treatment of fetal anomaly and related professional advice,” and “the choice of the pregnant woman and her family.” Our findings are consistent with a French study in which perception of disease severity is the greatest predictor of the acceptability of TOPFA (19). Health professionals in Israel also believe that acceptance of the termination of pregnancy option is related to the type and nature of the abnormality diagnosed (18).

### 4.3. Attitudes of health professionals toward family member’s decision-making rights on TOPFA

The majority of health professionals believe that the decision regarding TOPFA should be made jointly by the couple, followed by the pregnant woman, and then the family. The decision to terminate a pregnancy after the discovery of fetal abnormalities is a difficult one and is often made under time pressure (31). Our findings are consistent with the French obstetricians, who also believe that the decision to terminate a pregnancy should be a parental choice (19). In the United Kingdom, fetal abnormalities are recognized as grounds for allowing termination of pregnancy under Clause C of the current legislation, which permits legal termination of pregnancy before 24 weeks of gestation if continuation of the pregnancy would affect the mental health of the mother (20).

The findings of our study show that a small percentage of Chinese health professionals believe that joint decision-making should be used for TOPFA. This is consistent with Turkish physicians, who also believe that the entire family should have the right to decide on TOPFA (23). In traditional Chinese culture, families play an important role in medical decisions and may even make decisions on behalf of the patient (32). Given the complexity of the situation surrounding a fetus with a birth defect, joint decision-making by the parents or family members is a reasonable approach.

### 4.4. Factors influencing the health professionals’ attitudes toward TOPFA

Regarding TOPFA, numerous factors, encompassing personal viewpoints, professional directives, and institutional frameworks, significantly influence the decision-making process among health professionals working in fetal medicine clinics (33). Religious beliefs and training were found to be associated with health professionals’ attitudes toward TOPFA toward congenital heart disease in Case 1. Our findings are consistent with research in the UK (34), France (19) and Israel (35), health professionals with religious beliefs were more likely to oppose the termination of pregnancy of the fetus, possibly due to interpreting the issue of the fetus’s right to life through the norms of their own religious beliefs. For instance, Catholicism prohibits abortion, and therefore, Catholics may be more likely to respect the fetus’ right to life (36). Additionally, religious affiliation has been shown to be significantly related to opinions about abortion and its complexity (37). On the other hand, training related to birth defect prevention and control has been found to increase health professionals’ awareness of the importance of birth defect surveillance (38). Professionals who have received such training are more knowledgeable about the treatment of fetuses with defects and may be more inclined to accept babies with congenital defects once they are born.

Health professionals aged 51 and above 51 were more likely to oppose the termination of pregnancy of a fetus with cleft lip and palate in Case 2. This may be due to their greater experience with cases related to cleft lip and palate and their deeper knowledge of this condition, which could increase their confidence in the rehabilitation and treatment of fetuses with this malformation. Health professionals with intermediate-level professional titles were more likely to support the termination of pregnancy of a fetus with cleft lip and palate in Case 2 compared with professionals without titles or temporary hires. This could be attributed to differences in the professional training received by professionals at different levels, as well as the range of birth defect cases they encounter. Health professionals with intermediate-level professional titles may draw upon their own work experience to make judgments about the fetal right to life and may make different choices depending on the specific situation. In contrast, temporary employees may lack relevant birth defect prevention and control experience and may base their judgments on their own life experience. In contrast to full-time health professionals, part-time health professionals support the termination of pregnancy of the fetus with cleft lip and palate in Case 2. This may be due to their limited experience working with birth defects and their unfamiliarity with the treatment of fetuses with cleft lip and palate, which could make them more cautious about decisions regarding whether fetuses with this birth defect should be born.

Minority and married health professionals were found to have a higher likelihood of supporting the termination of pregnancy of a fetus with phenylketonuria in Case 4. It is possible that these health professionals took into account the fact that children with phenylketonuria require ongoing care from their families and that treatment options can vary widely between individuals (27). National customs can also influence perinatal practices in different countries (39). Health professionals working in clinical service departments and maternal and child health departments were more likely to oppose the termination of pregnancy of a fetus with phenylketonuria in Case 4. This could be attributed to their greater familiarity with the basic conditions of birth defect diseases and related professional knowledge, as well as their exposure to a larger number of children with birth defects in their daily work. As such, they may rely more heavily on their professional knowledge and experience when making decisions regarding fetuses with birth defects.

### 4.5. Limitations

Our study was subject to several limitations, as it was conducted solely among healthcare professionals, thereby potentially inclining the findings toward a medically-driven perspective on the birth of fetuses with defects, introducing a certain degree of bias. Furthermore, our participant group was constrained to a single region and thus may not precisely reflect the sentiments of the wider population at a national level. The complexity of ethical considerations accompanying the practical execution of birth defect prevention and control, though not addressed in this study, necessitates further exploration in subsequent studies.

## 5. Conclusion

Our findings indicate that healthcare professionals exhibit variability in their attitudes toward TOPFA based on the type of birth defect. There is a general consensus that the key factors guiding the decision to support TOPFA include the potential impact of fetal anomalies on fetal function and development, with the decision ideally made jointly by the couple. Influential factors such as religious beliefs, professional training, age, and job title can significantly shape the attitudes of healthcare professionals toward TOPFA. The results of this study may inform the development of guidelines for the prevention and treatment of birth defects. Future research should expand to compare the attitudes of different stakeholder groups toward TOPFA, such as pregnant women and their family members. It is critical to delve deeper into the unique challenges each population encounters.

## Data availability statement

The original contributions presented in the study are included in the article/Supplementary material, further inquiries can be directed to the corresponding authors.

## Author contributions

YW and YL conducted the literature review, participated in distributing the questionnaire and writing the first draft, and conducted the statistical analysis. XW, XZ, and YZ helped to revise the article. DL and XL designed and conceived this research. All authors read and approved the final manuscript.

## Funding

This present study was financially supported by Major Program of National Social Science Fund of China, Research on Moral Issues in the Field of Contemporary Science and Technology (22&ZD044); by Changsha Natural Science Foundation, Research on Quality Assessment of Ethics Committees in Medical and Health Institution (kq2202362); by the China Postdoctoral Science Foundation (2023M733977); by the Management Research Fund of Xiangya Hospital, Central South University (2021GL13); by the Fundamental Research Funds for the Central Universities of Central South University (2023ZZTS0522); by Major Scientific and Technological Projects for collaborative prevention and control of birth defects in Hunan Province (2019SK1010).

## Conflict of interest

The authors declare that the research was conducted in the absence of any commercial or financial relationships that could be construed as a potential conflict of interest.

## Publisher’s note

All claims expressed in this article are solely those of the authors and do not necessarily represent those of their affiliated organizations, or those of the publisher, the editors and the reviewers. Any product that may be evaluated in this article, or claim that may be made by its manufacturer, is not guaranteed or endorsed by the publisher.
