# Supplementary material for: Attitudes of Chinese maternal and child health professionals toward termination of pregnancy for fetal anomaly: a cross-sectional survey
Source: Front Public Health. 2023 Sep 7;11:1189266. doi: 10.3389/fpubh.2023.1189266 (PMC10513408; doi:10.3389/fpubh.2023.1189266)
Supplement: Supplementary file 1 [file Table_1.DOCX]

**Appendix: Health Professionals’ Attitudes toward Termination of Pregnancy for Fetal Anomaly (TOPFA)**

**Case 1:**

Mrs. Wang and her husband are both ordinary urban residents with regular incomes. Mrs. Wang had a previous pregnancy experience, but the fetus miscarried due to congenital defects. Now, at the age of 31, she is pregnant again, and the whole family values this fetus very much. At 23 weeks of pregnancy (6 months gestation), an ultrasound examination revealed that the fetus had severe congenital heart disease. After further examination, the doctor informed Mr. and Mrs. Wang that this type of congenital heart disease can be cured through multiple repair surgeries after the baby is born, with a total estimated cost of about one hundred thousand RMB or higher. After consulting multiple experts, although they were still hesitant, Mr. and Mrs. Wang have expressed their intention to keep the child.

**Question (1-4):**

1. If the decision is up to you, would you consent to the termination of the pregnancy in these cases? [Note: Please choose an option regardless of your considerations.]

(1) Disagree (2) Agree.

1. What are the main factors that influence your judgment on whether the fetus in the above case "should" or "should not" be born? [Note: ① Please choose from the following factors and you can select multiple ones; ② After selecting, please rank these factors according to their importance: You can input numbers in parentheses (the smaller the number, the higher the importance, for example: the number "1" represents the highest importance); ③ If there are other considerations that are not listed, please fill them in on the blank line.]

( ) Religious or belief considerations

( ) The fetus is a life that should be preserved

( ) Autonomous choice of pregnant women and their families

( ) Severity of the fetus's disease

( ) The impact of the fetus's disease on the development of body functions and growth

( ) The adverse effects of the fetus's condition on the pregnant woman

( ) The negative impact of the fetus's condition on the family or a family member

( ) Acceptance level of the fetal disease by the family

( ) Clinical professional assessment of treatment indications for fetal diseases and relevant professional advice

( ) Impact on fairness in resource allocation such as medical/social security

( ) Regardless of the severity of the fetal defects and the family's financial situation, parents should take responsibility for their own children

( ) Other 1________________________________

( ) Other 2________________________________

|  | **Strongly disagree** | **disagree** | **uncertain** | **agree** | **strongly agree** |
| --- | --- | --- | --- | --- | --- |
| 3. From your personal perspective, do you agree or disagree with Mr. and Mrs. Wang's willingness to keep the child? |  |  |  |  |  |

4. In this case, who do you think has the right to make the final decision on whether to continue or terminate the pregnancy of a fetus with birth defects? [Note: Single choice]

(1) Mrs. Wang (2) Mr. Wang (3) Both Mr. and Mrs. Wang (4) Joint decision-making by family.

**Case 2:**

Mrs. Wu is a rural resident who, during 22-24 weeks of pregnancy (around 6 months gestation), was diagnosed with a cleft lip and palate in the fetus through prenatal ultrasound examination. No other abnormalities were found. After consulting health professions. Mrs. Wu learned that this condition can be repaired through surgery, but the recovery effect after surgery is difficult to determine at present. After fully understanding the relevant information, Mr. and Mrs. Wu are willing to give birth to the child. However, the child's grandparents believe that even if the cleft lip and palate can be repaired, it may affect some of the child's functions in the future and can be visibly noticed on the appearance. They are inclined towards wanting Mrs. Wu to have an abortion for fear that the child will be subjected to gossip from the villagers in the future.

**Question (1-5):**

1. If the decision is up to you, would you consent to the termination of the pregnancy in these cases? [Note: Please choose an option regardless of your considerations.]

(1) Disagree (2) Agree.

2. Based on the previous question, what are the factors to consider whether the fetus in the above case should or should not be born? [Note: ① Please choose from the following factors and you can select multiple ones; ② After selecting, please rank these factors according to their importance: You can input numbers in parentheses (the smaller the number, the higher the importance, for example: the number "1" represents the highest importance); ③ If there are other considerations that are not listed, please fill them in on the blank line.]

( ) Religious or belief considerations

( ) The fetus is a life that should be preserved

( ) Autonomous choice of pregnant women and their families

( ) Severity of the fetus's disease

( ) The impact of the fetus's disease on the development of body functions and growth

( ) The adverse effects of the fetus's condition on the pregnant woman

( ) The negative impact of the fetus's condition on the family or a family member

( ) Acceptance level of the fetal disease by the family

( ) Clinical professional assessment of treatment indications for fetal diseases and relevant professional advice

( ) Impact on fairness in resource allocation such as medical/social security

( ) Regardless of the severity of the fetal defects and the family's financial situation, parents should take responsibility for their own children

( ) Other 1 ________________________________

( ) Other 2 ________________________________

|  | **Strongly disagree** | **disagree** | **uncertain** | **agree** | **strongly agree** |
| --- | --- | --- | --- | --- | --- |
| 3. From your personal perspective, do you agree or disagree with Mr. and Mrs. Wu's decision to give birth to the child? |  |  |  |  |  |
| 4. From your personal perspective, do you agree or disagree with the child's grandparents' decision to have Mrs. Wu undergo an abortion? |  |  |  |  |  |

5. In this case, who do you think has the right to make the final decision on whether to continue or terminate the pregnancy of a fetus with birth defects? [Note: Single choice]

1. Mrs. Wu (2) Mr. Wu (3) Both Mr. and Mrs. Wu (4) The child's grandparents

(5) Joint decision-making by family.

**Case 3:**

Mrs. Yang underwent a B-ultrasound examination at 18 weeks of pregnancy (more than 4 months gestation), which showed that the middle three fingers of the fetus's right hand were missing, and no other abnormalities were found. After a comprehensive examination, the doctor informed Mrs. Yang and her husband that it is currently impossible to evaluate the prognosis of the child. Considering that the child may face lifelong disability and social discrimination after birth, Mr. and Mrs. Yang initially planned to have an induced abortion. However, the child’s grandparents believed that it was too pitiful for the baby to be aborted because of the disability caused by missing fingers, and insisted that the baby can be born. Therefore, they repeatedly asked Mr. and Mrs. Yang to consider their decision carefully.

**Question (1-5):**

1. If the decision is up to you, would you consent to the termination of the pregnancy in these cases? [Note: Please choose an option regardless of your considerations.]

(1) Disagree (2) Agree.

2. Based on the previous question, what are the factors to consider whether the fetus in the above case should or should not be born? [Note: ① Please choose from the following factors and you can select multiple ones; ② After selecting, please rank these factors according to their importance: You can input numbers in parentheses (the smaller the number, the higher the importance, for example: the number "1" represents the highest importance); ③ If there are other considerations that are not listed, please fill them in on the blank line.]

( ) Religious or belief considerations

( ) The fetus is a life that should be preserved

( ) Autonomous choice of pregnant women and their families

( ) Severity of the fetus's disease

( ) The impact of the fetus's disease on the development of body functions and growth

( ) The adverse effects of the fetus's condition on the pregnant woman

( ) The negative impact of the fetus's condition on the family or a family member

( ) Acceptance level of the fetal disease by the family

( ) Clinical professional assessment of treatment indications for fetal diseases and relevant professional advice

( ) Impact on fairness in resource allocation such as medical/social security

( ) Regardless of the severity of the fetal defects and the family's financial situation, parents should take responsibility for their own children

( ) Other 1 ________________________________

( ) Other 2 ________________________________

|  | **Strongly disagree** | **disagree** | **uncertain** | **agree** | **strongly agree** |
| --- | --- | --- | --- | --- | --- |
| 3. From your personal perspective, do you agree or disagree with Mr. and Mrs. Yang's decision to give up the fetus? |  |  |  |  |  |
| 4. From your personal perspective, do you agree or disagree with the child's grandparents' belief that the child can be born? |  |  |  |  |  |

5. In this case, who do you think has the right to make the final decision on whether to continue or terminate the pregnancy of a fetus with birth defects? [Note: Single choice]

(1) Mrs. Yang (2) Mr. Yang (3) Both Mr. and Mrs. Yang (4) The child's grandparents (5) Joint decision-making by family.

**Case 4:**

Mrs. Duan was diagnosed with possible Phenylketonuria (PKU) in the fetus during prenatal examination at 16 weeks of pregnancy (4 months gestation). The doctor informed her that this disease may cause growth and developmental delays, as well as neurological and psychological issues in the child. However, if diagnosed early and treated properly, the fetus can grow and develop normally and maintain a normal life in adulthood. Children with PKU cannot break down certain proteins normally, so they need lifelong treatment through diet control and medication (for example, the children need to eat specific foods and take medicine every day to achieve balanced nutrition). After being fully informed by the doctor about the disease, Mrs. Duan's husband believed that they should have an induced abortion, while Mrs. Duan thought that although it would bring a significant financial burden, the child could be kept stable through active treatment, so she tended to keep the fetus.

**Question (1-5):**

1. If the decision is up to you, would you consent to the termination of the pregnancy in these cases? [Note: Please choose an option regardless of your considerations.]

(1) Disagree (2) Agree.

2. Based on the previous question, what are the factors to consider in determining whether the fetus in the above case should or should not be born? [Note: ①Please select from the listed factors below; multiple selections are allowed. ②After making your selection, please rank these factors according to their importance by putting a number in front of the options (the smaller the number, the more important it is. For example, the number "1" indicates the highest importance). ③If there are other factors to consider that are not listed, please add them on the blank lines.]

( ) Religious or belief considerations

( ) The fetus is a life that should be kept

( ) The autonomy of the pregnant woman and her family's choice

( ) The severity of the fetus's own disease

( )The impact of the fetus's disease on the body function and growth and development

( ) The adverse effects of the fetus's disease on the pregnant woman

( ) The adverse effects of the fetus's disease on the family or a family member

( ) The degree to which the family accepts the fetus's disease

( ) Clinical professionals' assessment of treatment indications and related professional advice for the fetus's disease

( ) The impact on the fairness of resource allocation for medical/social security, etc.

( ) Regardless of the degree of the fetus's defect and the family's financial status, one should take responsibility for their own child

( ) Other 1 ________________________________

( ) Other 2 ________________________________

|  | **Strongly disagree** | **disagree** | **uncertain** | **agree** | **strongly agree** |
| --- | --- | --- | --- | --- | --- |
| 3. From your personal perspective, do you agree or disagree with Mrs. Duan's decision to keep the child? |  |  |  |  |  |
| 4. From your personal perspective, do you agree or disagree with Mr. Duan's decision not to want the child? |  |  |  |  |  |

5. In this case, who do you think has the right to make the final decision on whether to continue or terminate the pregnancy of a fetus with birth defects:

(1) Ms. Duan (2) Mr. Duan (3) Both Mr. and Mrs. Duan (4) Joint decision-making by family
